# *Klebsiella pneumoniae* pyogenic liver abscess mimicking hepatic malignancy in a non-diabetic patient: A case report from Kazakhstan

**DOI:** 10.1016/j.radcr.2026.09.056

**Published:** 2026-10-15

**Authors:** Gaukhar Medetova, Davranbek Abduvaliyev, Arailym Kizayeva, Ayaulym Gabbassova, Indira Bozheyeva

**Affiliations:** Scientific Research Institute of Radiology named after Zh. H. Khamzabayev, Astana Medical University, Beibitshilik 49A, Astana, 010000, Kazakhstan

**Keywords:** *Klebsiella pneumoniae*, Liver abscess, Differential diagnosis, CT, MRI, Non-diabetic

## Abstract

Pyogenic liver abscess is an infectious condition associated with significant morbidity and mortality, with *Klebsiella pneumoniae* emerging as a major pathogen. We report a case of *Klebsiella pneumoniae* pyogenic liver abscess in a 76-year-old woman from Kazakhstan, representing one of the first indexed reports from Central Asia. The patient presented with prolonged fever, jaundice, and constitutional symptoms without diabetes mellitus. Initial ultrasound revealed a large heterogeneous hepatic mass with a solid, tumor-like appearance, raising concern for malignancy in the setting of chronic hepatitis B infection. MRI and contrast-enhanced CT demonstrated diffusion restriction, multiloculated architecture, and mild peripheral and septal enhancement without arterial hyperenhancement or venous washout, favoring hepatic abscess over hepatocellular carcinoma. The solid, tumor-like ultrasound appearance, atypical for a pyogenic abscess in its resemblance to hepatic malignancy, posed the primary diagnostic challenge, underscoring the value of multimodal imaging in characterizing hepatic lesions. Additionally, KPLA should be considered in the differential diagnosis even in the absence of classical metabolic risk factors and in regions with limited epidemiological data.

## Introduction

Liver abscesses are rare but life-threatening complications of bacterial, fungal, protozoal and worm infections [1]. Among these, pyogenic liver abscess (PLA) is a critical infectious condition characterized by the formation of purulent collections within the hepatic parenchyma as a result of bacterial invasion, associated with significant morbidity and mortality [2]. Common causative organisms include *Escherichia coli, Streptococcus* spp., anaerobes, and *Klebsiella pneumoniae* [3], the latter being the predominant pathogen in Asian populations [3,4] and an increasingly recognized cause in Western countries [4,5]. Geographical variation in incidence is well documented, with substantially higher rates reported in Asian populations compared with Western countries [6,7].

*Klebsiella pneumoniae* pyogenic liver abscess (KPLA) is of clinical concern, as it frequently presents with a solid, tumor-like imaging appearance that poses significant diagnostic challenges and carries the risk of being misclassified as hepatic malignancy [8]. To the best of our knowledge, based on a search of PubMed and Scopus, no indexed case reports of KPLA from Kazakhstan or Central Asia have been previously identified. While large-scale epidemiological data on PLA have been generated predominantly from East Asian and Western populations [2], no population-based studies from Central Asia have been identified in these databases, reflecting a broader gap in the global understanding of KPLA's geographic distribution. Reporting cases from this region is therefore important for expanding the epidemiological landscape of KPLA beyond traditionally studied populations.

Herein, we report a case of KPLA in a 76-year-old woman from Kazakhstan, in whom multimodal imaging played a pivotal role in establishing the diagnosis prior to drainage and microbiological confirmation, highlighting the diagnostic challenges posed by the tumor-like radiological presentation of KPLA [8] and the importance of maintaining clinical suspicion regardless of metabolic risk factors.

## Case presentation

A 76-year-old woman was admitted to the general surgery department in Astana, Kazakhstan, with complaints of transient pain in the right upper quadrant, nausea, vomiting, and loss of appetite with early satiety for the past ten days, as well as prolonged subfebrile temperature of 37.2°C–37.6°C for three weeks, and a persistent temperature of 39.0°C for two days. The patient's comorbidities included chronic hepatitis B virus (HBV) infection and gallbladder polyps. The HBV infection was identified incidentally during the current admission, with no prior diagnosis or antiviral therapy. No clinical or biochemical features of hepatic decompensation were present at the time of admission. Family history was unremarkable. Physical examination revealed jaundice of the skin and sclera.

Laboratory investigations revealed marked leukocytosis (24.92 × 10⁹/L) with a left shift, thrombocytopenia (136 × 10⁹/L), elevated inflammatory markers, hypoproteinemia, and biochemical evidence of hepatic dysfunction including elevated transaminases, alkaline phosphatase, and total bilirubin. Tumor markers (CA 19-9, CEA, and alpha-fetoprotein) were within normal limits, and fasting glucose was normal at 4.04 mmol/L, excluding diabetes mellitus. Specifically, leukocytosis was 24.92 × 10⁹/L with band neutrophils of 7% and neutrophilia of 85%, thrombocytopenia was 136 × 10⁹/L, and erythrocyte sedimentation rate was 45 mm/h (Panchenko) and 57 mm/h (Westergren). Total protein was reduced at 55.60 g/L (reference 64.0–83.0), urea was elevated at 18.40 mmol/L, creatinine at 169.00 μmol/L, alanine aminotransferase at 56.30 U/L, aspartate aminotransferase at 72.20 U/L, alkaline phosphatase at 337.0 U/L, and total bilirubin at 74.10 μmol/L. Tumor markers (CA 19-9 24.3 ME/mL, CEA 0.64 ng/mL, alpha-fetoprotein < 0.9 ng/mL) were all within normal limits.

Abdominal ultrasound revealed a poorly defined, heterogeneous mass in the left lobe of the liver, affecting the second, third, and fourth segments, measuring 12.3 × 7.0 × 9.0 cm and showed mixed echogenicity with internal anechoic areas with mixed internal vascularization without a dominant arterial feeding vessel (Figs. 1A and 1B).

**Fig. 1:**
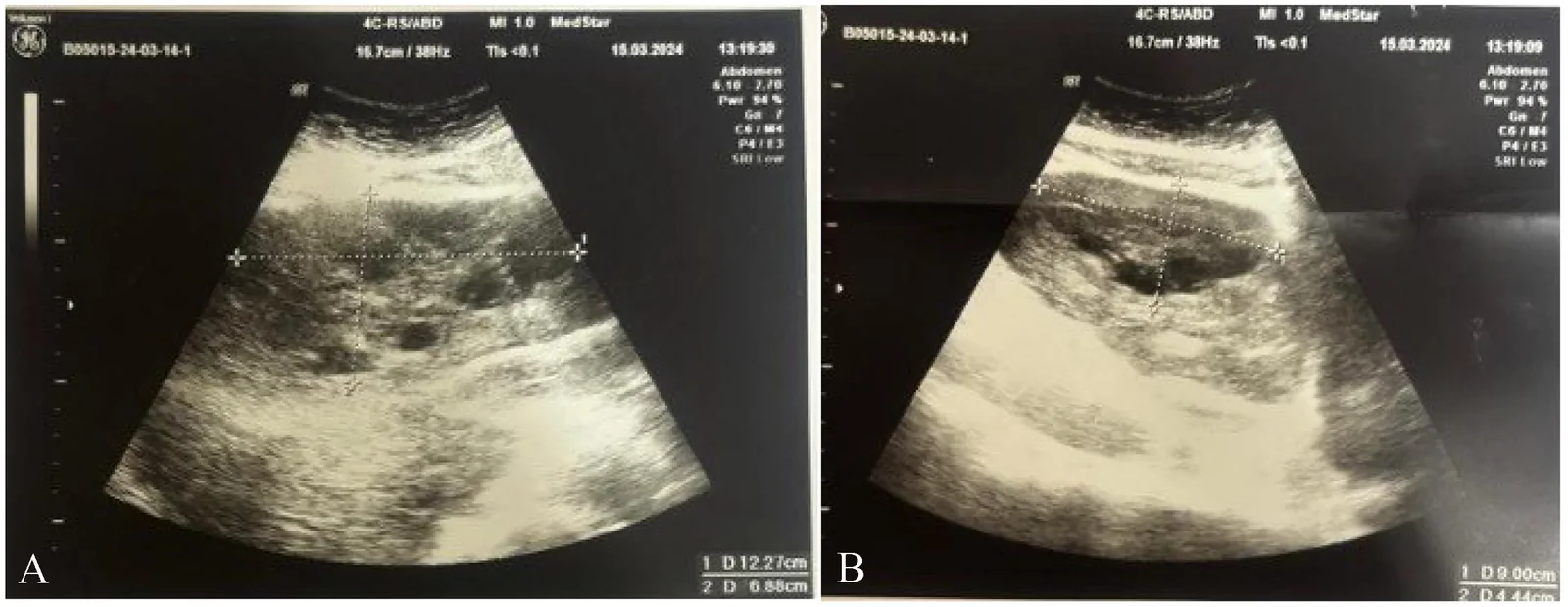
Abdominal ultrasound. (A) B-mode ultrasound, longitudinal view. A poorly defined, heterogeneous mass occupying segments II–IV of the left hepatic lobe, measuring 12.27 × 6.88 cm, with mixed echogenicity and internal anechoic areas, raising concern for hepatic malignancy on initial imaging. (B) B-mode ultrasound, transverse view. An additional cross-sectional view of the lesion (9.00 cm), demonstrating irregular, ill-defined margins with heterogeneous internal architecture.

MRI demonstrated a heterogeneous multiloculated mass involving segments II, III, and IV of the left hepatic lobe with irregular, ill-defined margins and hyperintense internal septations on T2-weighted fat-suppressed sequences. T1-weighted contrast-enhanced imaging revealed mild peripheral and septal enhancement without arterial hyperenhancement or venous washout, incompatible with hepatocellular carcinoma. Diffusion-weighted imaging (b = 800 s/mm²) demonstrated restricted diffusion of the intracavitary contents, consistent with increased viscosity of purulent material (Figs. 2A–E).

**Fig. 2:**
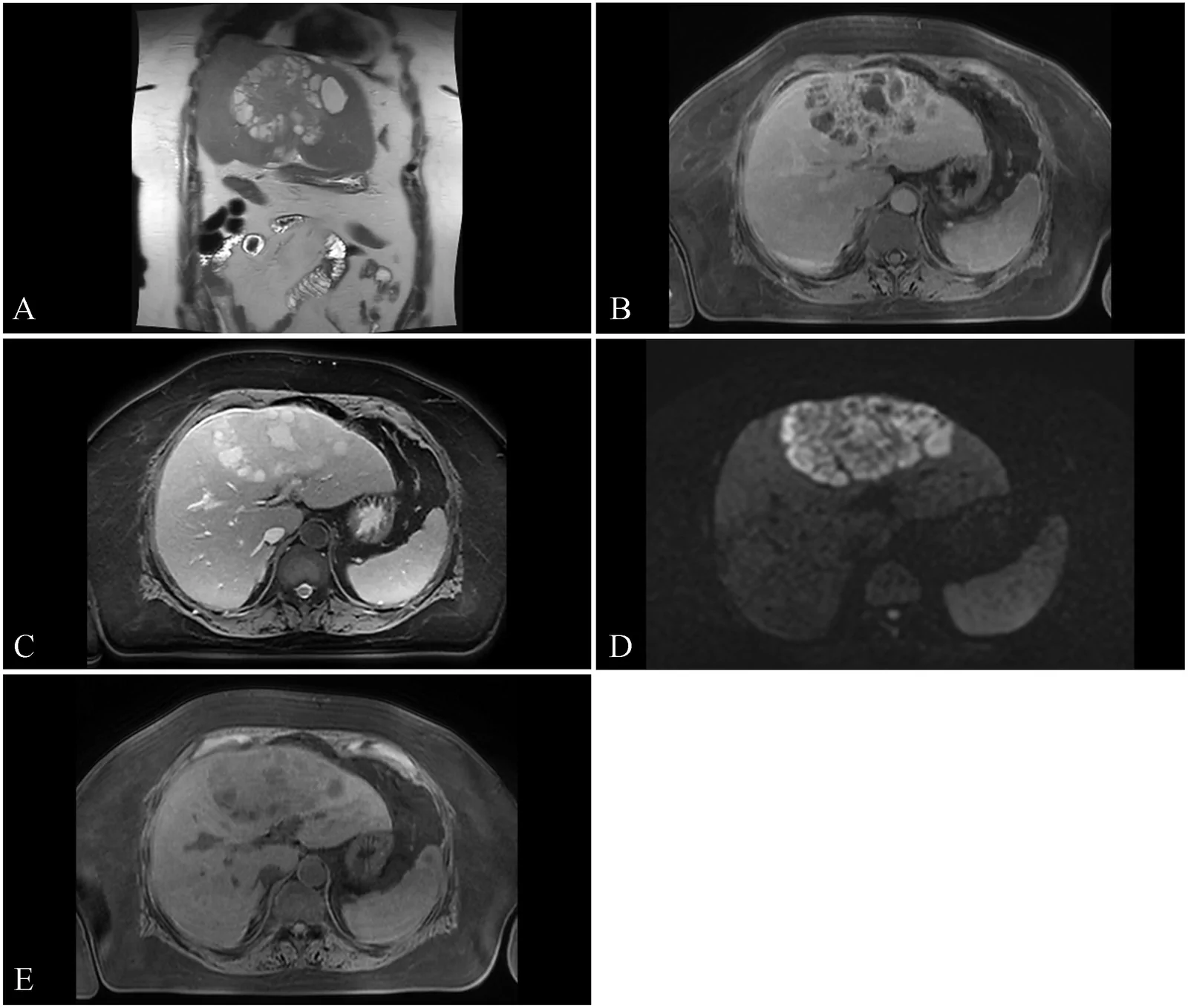
Magnetic resonance imaging (MRI) of the liver. (А) Coronal T2-weighted image. Multiloculated internal architecture of the lesion with craniocaudal extent, demonstrating involvement of segments II, III, and IV. (B) Axial T2-weighted fat-suppressed image. Heterogeneous mass with hyperintense internal septations and irregular ill-defined margins involving segments II and IV. (C) Axial T1-weighted image. Hypointense mass within the left hepatic lobe with irregular margins. (D) Axial T1-weighted contrast-enhanced image. Mild peripheral and septal enhancement without arterial hyperenhancement or venous washout, incompatible with hepatocellular carcinoma. (E) Diffusion-weighted image (b = 800 s/mm²). Persistent hyperintensity of the intracavitary contents indicating restricted diffusion, consistent with increased viscosity of purulent material rather than tumor cellularity.

Diffusion-weighted imaging (b = 800 s/mm²) demonstrated restricted diffusion of the intracavitary contents, consistent with increased viscosity of purulent material (Fig. 2E).

Non-contrast abdominal CT identified a multiloculated, predominantly solid mass within the second, fourth, and eighth hepatic segments. Contrast-enhanced CT in the arterial phase demonstrated mild peripheral and septal enhancement without central enhancement, a pattern consistent with a hepatic abscess (Figs. 3A–C).

**Fig. 3:**
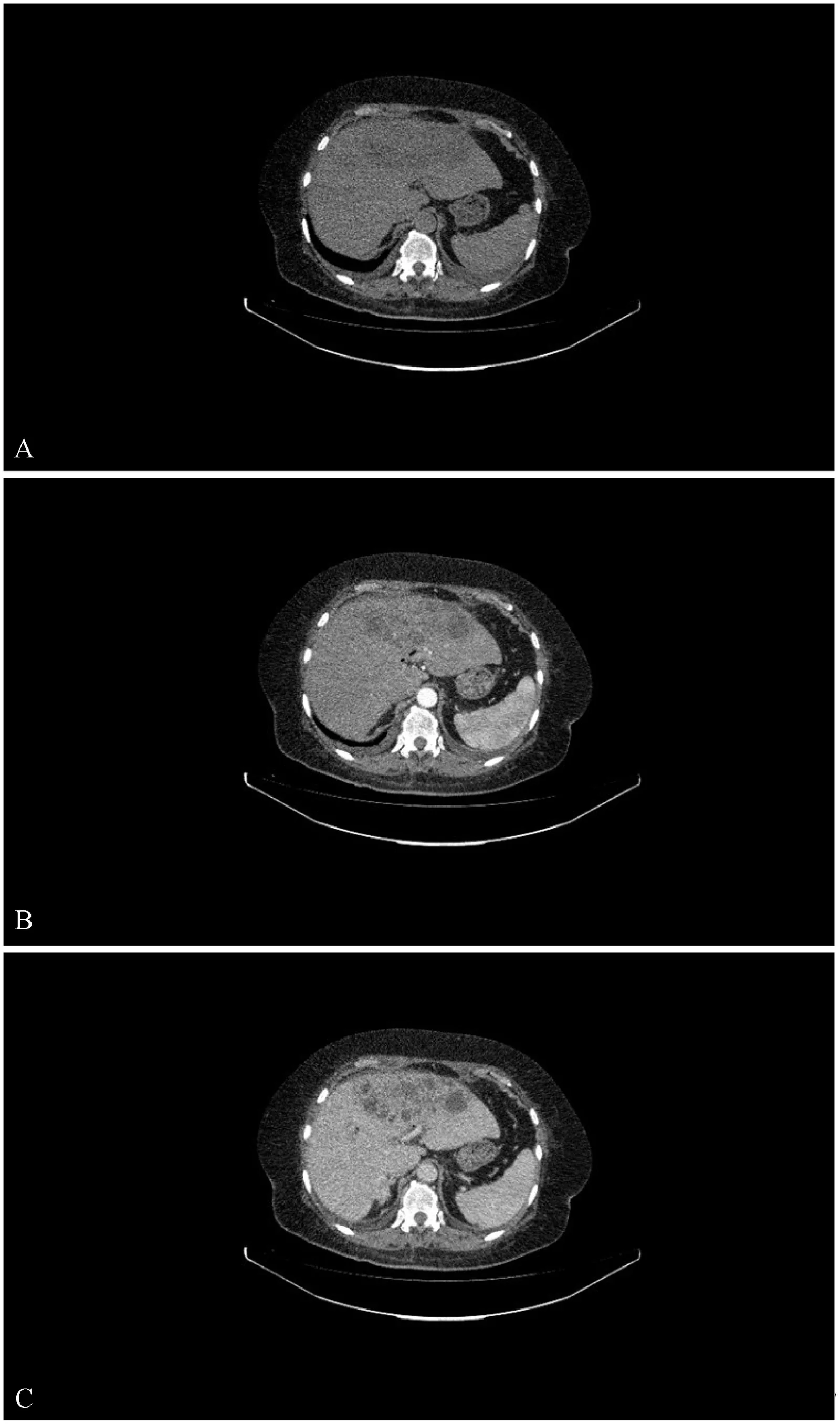
Computed tomography (CT) of the abdomen. (A) Non-contrast CT, axial view. Multiloculated, predominantly solid hypodense mass within hepatic segments II, IV, and VIII, with internal septations. (B) Contrast-enhanced CT, arterial phase, axial view. Mild peripheral and septal enhancement without central enhancement or arterial hyperenhancement, favoring hepatic abscess over hepatocellular carcinoma. (C) Contrast-enhanced CT, portal venous phase, axial view - absence of venous washout.

Chest CT revealed focal parenchymal opacities in segments 3 and 4 of the left lung, consistent with early septic embolic foci in the context of confirmed *Klebsiella pneumoniae* bacteremia — a recognized metastatic complication of KPLA. No cavitation, pleural effusion, or consolidation was identified (Figs. 4A–D).

**Fig. 4:**
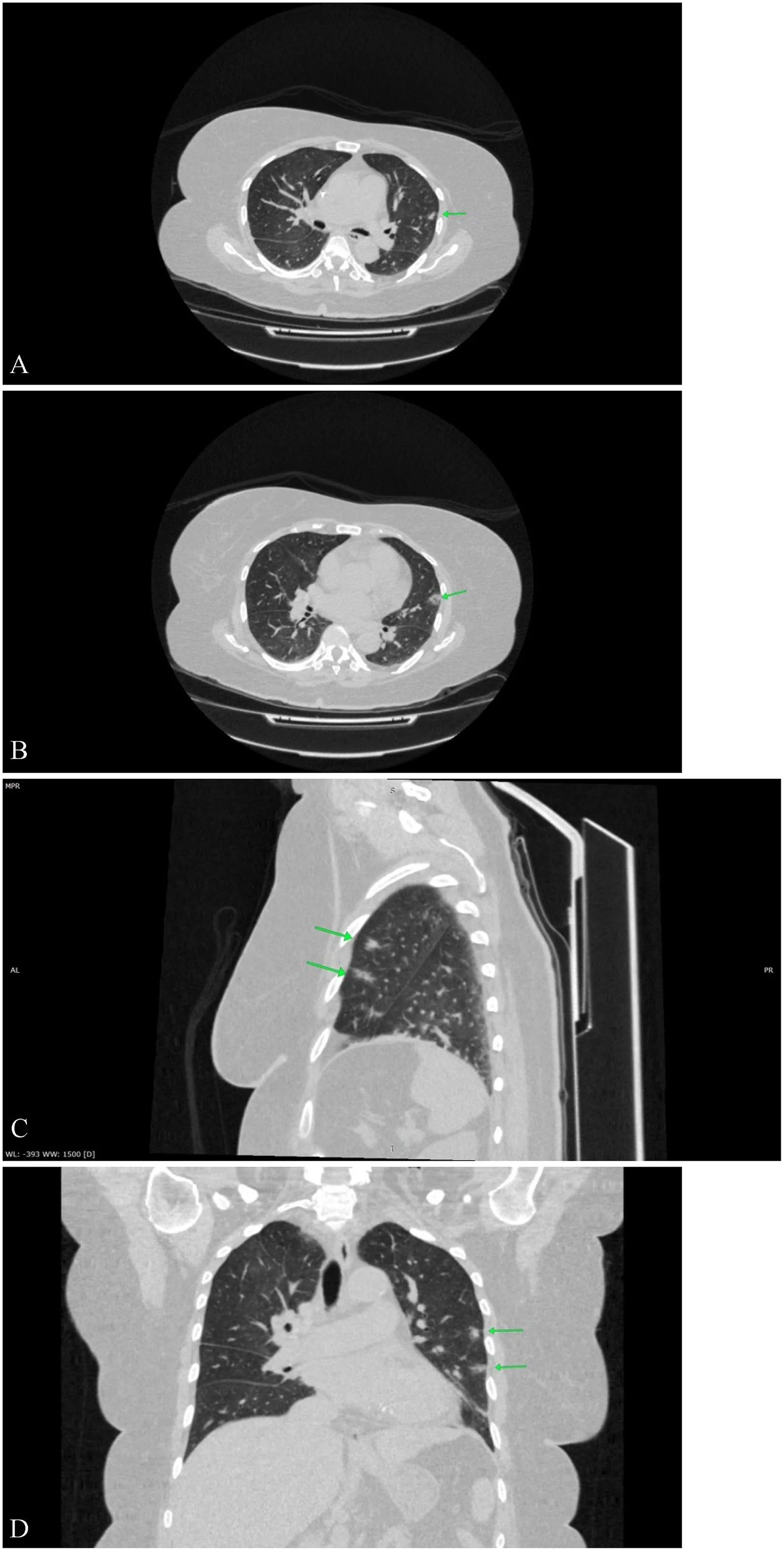
Chest computed tomography (CT) of lung. (A) Axial CT, lung window. Focal parenchymal opacity in segment 3 of the left lung, without cavitation or pleural effusion, consistent with early septic embolic focus in the context of confirmed *Klebsiella pneumoniae* bacteremia (arrow). (B) Axial CT, lung window. Focal parenchymal opacity in segment 4 of the left lung, without cavitation or pleural effusion (arrow). (C) Coronal CT, lung window. Both focal parenchymal opacities in the left lung demonstrated simultaneously, confirming their location in segments 3 and 4 (arrows). (D) Sagittal CT, lung window. Sagittal reconstruction demonstrating both focal parenchymal opacities in the left lung without cavitation or consolidation (arrows).

Upon admission, empirical intravenous antibiotic therapy was initiated with ceftriaxone (Цеф III [Cef III], Kazakhstan) and metronidazole (Метри [Metride], Kazakhstan), providing broad-spectrum coverage against gram-negative bacilli and anaerobic organisms pending microbiological confirmation. Percutaneous ultrasound-guided drainage of the abscess was subsequently performed, and culture of the drained material identified *Klebsiella pneumoniae*. Intravenous therapy was continued for ten days until clinical stabilization, after which the patient was transitioned to oral ciprofloxacin (500 mg twice daily) (Амоксициллин (Ciprofloxacin, Kazakhstan) for a further fourteen days, yielding a total antibiotic treatment duration of twenty-four days. Follow-up instructions included hepatology and infectious disease consultations, as well as hepatobiliary and pancreatic ultrasound surveillance every six months.

## Discussion

Pyogenic liver abscess (PLA) is a suppurative, space-occupying lesion of the hepatic parenchyma, arising most commonly via ascending biliary tract infection, portal vein bacteremia, or hematogenous spread [1]. Other etiological types include amoebic and fungal liver abscess, though PLA predominates in developed countries and accounts for most hepatic abscess cases [1,2]. Despite advances in diagnostic imaging and antimicrobial therapy, PLA remains associated with significant morbidity and mortality if not promptly recognized and treated [9].

Among the causative pathogens, *Klebsiella pneumoniae* has emerged as a pathogen of particular concern, capable of producing a distinct invasive syndrome characterized by a tumor-like hepatic mass and metastatic complications [8]. As data from Central Asia, including Kazakhstan, remain largely absent from the international literature [2], awareness of the clinical presentation and imaging features of KPLA is essential for timely diagnosis in non-endemic regions.

Despite its predominance in Asian populations, *Klebsiella* is the causative agent for approximately only 10% of pyogenic liver abscess cases in European settings [10,11]. As such KPLA must also be considered in patients from regions with intermediate epidemiological profiles, such as Central Asia.

Reporting cases from such underrepresented regions contributes to a more complete global understanding of KPLA’s epidemiological distribution.

The clinical presentation of our patient was consistent with reported manifestations of KPLA, including prolonged low-grade fever, jaundice, vomiting, and generalized weakness [12,13]. Notably, right upper quadrant pain, which is observed in the majority of KPLA cases was not reported here. The left lobe predominance of the abscess, involving segments II, III, and IV, is also less typical, as KPLA more frequently affects the right hepatic lobe [9]. Laboratory investigations corroborated an acute bacterial infectious process, demonstrating marked leukocytosis, thrombocytopenia, elevated inflammatory markers, and biochemical evidence of hepatic dysfunction including raised transaminases, alkaline phosphatase, and total bilirubin — all consistent with previously described laboratory profiles of KPLA [14]. Importantly, fasting glucose was within normal limits, confirming the absence of diabetes mellitus, which is reported as a comorbidity in 50–75% of KPLA cases [4,9]. Additionally, tumor markers including alpha-fetoprotein, CEA, and CA 19-9 were within normal limits, which, in the context of underlying chronic hepatitis B infection, was important in excluding hepatocellular carcinoma from the differential diagnosis. The absence of classic right upper quadrant pain, combined with the absence of diabetes mellitus, contributed to initial diagnostic uncertainty.

These clinical features were followed by an ultrasound finding that further supported the initial suspicion of hepatic malignancy, with a large, heterogeneous, poorly defined mass in the left hepatic lobe. Such a solid, tumor-like morphology represents a recognized diagnostic challenge in KPLA, which can present with an appearance distinct from the more typical cystic or multiloculated morphology of pyogenic abscesses caused by non-*Klebsiella* organisms [8,15]. This solid, tumor-like appearance (atypical for a pyogenic abscess in its resemblance to hepatic malignancy), together with the background of chronic hepatitis B virus infection—a recognized risk factor for hepatocellular carcinoma—favored hepatic malignancy in the initial differential diagnosis.

Subsequent MRI provided the first positive evidence in favor of an infectious etiology. Diffusion-weighted imaging demonstrated restricted diffusion characterized by persistent hyperintensity at higher b-values with corresponding low ADC signal, a finding reflecting the increased viscosity of intracavitary purulent contents — a pattern more consistent with abscess than with the restricted diffusion pattern characteristic of hypercellular solid malignancies [8]. Additionally, the absence of arterial phase hyperenhancement and venous washout on contrast-enhanced sequences was incompatible with hepatocellular carcinoma per EASL diagnostic criteria [16], effectively narrowing the differential.

Contrast-enhanced CT further characterized the lesion as a multiloculated, predominantly solid hepatic mass with mild peripheral and septal enhancement — a pattern consistent with the CT appearance of KPLA as described by Alsaif et al. [15], where solid or multiloculated morphology with septal enhancement is a recognized feature distinguishing KPLA from other pyogenic abscesses. The absence of arterial hypervascularity, washout, and capsule formation argued against a malignant etiology [16]. The integration of findings across all three modalities was pivotal in reclassifying the lesion from a suspected malignancy to a hepatic abscess prior to percutaneous drainage, which subsequently confirmed *Klebsiella pneumoniae* as the causative pathogen. The key imaging features distinguishing KPLA from other hepatic abscesses and hepatocellular carcinoma are summarized in Table 1.

**Table 1:** Comparative multimodal imaging characteristics of KPLA, non-Klebsiella pyogenic liver abscess, amoebic abscess, fungal abscess, and hepatocellular carcinoma.

Diagnosis	Ultrasound	CT	MRI	Key differentiating feature	Ref
KPLA	Solid, hypoechoic mass; may mimic hepatic tumour	Single, solid or multiloculated focal hepatic lesion; mild septal enhancement	T2 hyperintense centre; T1 hypointense; DWI restriction	Solid tumour-like appearance; monomicrobial	[8,15]
Bacterial PLA (non-Klebsiella)	Hypoechoic with septa and debris; ill-defined	Cluster sign; double target sign; perilesional transient perfusion disorder; occasional gas	T2 hyperintense centre; T1 hypointense; DWI restriction	Polymicrobial; cluster sign; biliary origin	[8]
Amoebic abscess	Hypoechoic; well-delineated; low-level internal echoes	Hypoattenuating (10–20 HU); thick enhancing peripheral capsule; double target sign	T1 hypointense; T2 hyperintense centre; peripheral capsule enhancement; T2 hyperintense peripheral rim	Unilocular; right lobe predominance; travel history	[8,17]
Fungal abscess	Wheel-within-wheel sign; bull's eye sign; uniformly hypoechoic nodule	Multiple small hypoattenuating lesions; miliary pattern	Markedly T2 hyperintense; T1 hypointense; moderate enhancement	Immunocompromised host; miliary pattern	[8,17]
Hepatocellular carcinoma	-	Arterial hyperenhancement; portal venous washout; enhancing capsule	Arterial hyperenhancement; portal venous washout; hepatobiliary phase hypo intensity	HBV/cirrhosis background; arterial hyperenhancement absent in KPLA	[16]

CT (computed tomography), MRI (magnetic resonance imaging).

The focal pulmonary parenchymal changes identified on chest CT warrant particular attention. In the context of confirmed *Klebsiella pneumoniae* bacteremia, these findings are consistent with early septic pulmonary embolic foci, which is a recognized metastatic complication of KPLA occurring via hematogenous spread [4,18]. The lungs represent the second most commonly affected distant site in KPLA-associated metastatic infection, following the eyes [19]. Chest CT findings of septic pulmonary embolism in KPLA include focal nodules, peripheral wedge-shaped opacities, and ground-glass attenuation, which may precede cavitation and frank abscess formation [18]. The absence of follow-up cross-sectional thoracic imaging in this case precludes definitive characterization of these pulmonary changes and determination of whether they represented true metastatic foci or resolved with systemic antibiotic therapy — a recognized limitation of the present report.

The patient was managed with empirical intravenous ceftriaxone and metronidazole, initiated prior to microbiological confirmation and consistent with current guideline recommendations for pyogenic liver abscess of undetermined etiology, providing broad-spectrum coverage against gram-negative bacilli and anaerobic organisms pending culture results [1,3]. Third-generation cephalosporins combined with metronidazole represent a recommended first-line empirical regimen for pyogenic liver abscess, reflecting resistance rates of up to 90% against ampicillin among Enterobacteriaceae [1,20]. Following culture confirmation of *Klebsiella pneumoniae*, metronidazole was discontinued, as it confers no additional benefit against this aerobic monomicrobial pathogen, and ceftriaxone monotherapy was continued as the definitive regimen [1,20]. Percutaneous ultrasound-guided drainage was performed as the primary source control measure, which remains the preferred interventional approach for pyogenic liver abscess amenable to drainage [1]. Intravenous antibiotic therapy was continued for ten days until clinical stabilization, after which the patient was transitioned to oral ciprofloxacin (500 mg twice daily) for a further fourteen days, yielding a total treatment duration of twenty-four days — consistent with current recommendations for a three to four week course [3]. Early step-down to oral ciprofloxacin following clinical stabilization is supported by the A-KLASS randomized controlled trial, which demonstrated non-inferiority of oral ciprofloxacin to continued intravenous ceftriaxone for *Klebsiella pneumoniae* liver abscess [21]. The patient demonstrated progressive clinical improvement with resolution of fever and was discharged in a stable, afebrile condition. Hepatology and infectious disease follow-up consultations were arranged, alongside hepatobiliary ultrasound surveillance every six months.

## Limitations

This report has several limitations. Molecular characterization of the *Klebsiella pneumoniae* isolate, including capsular serotyping and virulence gene profiling, was not performed, precluding determination of whether a hypervirulent strain was responsible — information that would have provided important microbiological and epidemiological context [4]. Additionally, no follow-up cross-sectional imaging was obtained after percutaneous drainage to document radiological resolution, which is recommended as part of standard post-treatment monitoring [1]. Finally, as a single case report, the findings cannot be generalized, and no conclusions can be drawn regarding the broader epidemiology of KPLA in Kazakhstan.

## Conclusion

We report a case of *Klebsiella pneumoniae* pyogenic liver abscess in a 76-year-old non-diabetic woman from Kazakhstan — to the best of our knowledge, one of the first such cases described from Central Asia in the indexed medical literature. This case illustrates the complementary and sequential roles of multimodal imaging in establishing the correct diagnosis. Initial abdominal ultrasound identified a solid, tumor-like hepatic mass and raised the primary diagnostic concern for malignancy, prompting further cross-sectional imaging. MRI provided the first positive evidence in favor of an infectious etiology: diffusion-weighted imaging demonstrated restricted diffusion consistent with viscous purulent contents, while the absence of arterial hyperenhancement and venous washout on contrast-enhanced sequences was incompatible with hepatocellular carcinoma per EASL criteria. Contrast-enhanced CT confirmed the multiloculated architecture with peripheral and septal enhancement, further supporting the diagnosis of hepatic abscess and guiding percutaneous drainage. Chest CT identified focal pulmonary parenchymal opacities raising concern for early septic embolic foci, prompting initiation of empirical broad-spectrum antibiotic therapy. This case underscores that a structured multimodal imaging approach — with ultrasound as the initial alert modality, MRI for tissue characterization and diffusion assessment, and CT for anatomical delineation and interventional planning — is essential in differentiating KPLA from hepatic malignancy, particularly in patients without classical metabolic risk factors and in regions with limited epidemiological data.

## Ethics approval

Local ethical approval for this case report was obtained from Astana Medical University. The case was managed in accordance with the principles of the Declaration of Helsinki.

## Author contributions

AK, IB: conceptualization, data collection, manuscript drafting. AM, DA: imaging interpretation, figure preparation, critical revision. GM: supervision, final approval of the manuscript. All authors have read and approved the final version of the manuscript.

## Patient consent

Written informed consent was obtained from the patient for publication of this case report and any accompanying imaging data.

## Appendix Supplementary materials

Image, application 1

Image, application 2
